# Time-Series Multi-Omics Reveal Metabolic Reprogramming, Oxidative Stress and Programmed Cell Death in *M. bovis*-Infected Bovine Mammary Epithelial Cells

**DOI:** 10.3390/microorganisms14102241

**Published:** 2026-10-03

**Authors:** Tingwei Wang, Manling Zhu, Zhiyong Wu, Xingxu Zhao, Liang Zhang

**Affiliations:** 1 College of Veterinary Medicine, Gansu Agricultural University, Lanzhou 730070, China; wtw980203@163.com (T.W.); wuzhiyong1808@163.com (Z.W.); 2 School of Pharmaceutical and Life Sciences, Jiujiang University, Jiujiang 332005, China; 6140135@jju.edu.cn; 3 College of Veterinary Medicine, Lanzhou University, Lanzhou 730000, China

**Keywords:** *Mycoplasmopsis bovis*, MAC-T cells, multi-omics, metabolic reprogramming, programmed cell death

## Abstract

*Mycoplasmopsis bovis* is a major causative agent of bovine mastitis and causes substantial economic losses in the dairy industry because of the chronic and refractory nature of the infection. This study aimed to determine how *M. bovis* PG45 infection dynamically alters host metabolism and gene expression over time and whether these changes are accompanied by progressive redox imbalance and enrichment of multiple programmed cell death pathways. Transcriptomic and untargeted metabolomic profiles were analyzed at 8, 24, 48, and 72 h post-infection using three biological replicates for RNA sequencing and six biological replicates for metabolomic analysis. RNA-seq identified 779, 1777, 4631, and 4855 differentially expressed genes (DEGs) at 8, 24, 48, and 72 h, respectively, revealing a progressive expansion of transcriptional responses. KEGG enrichment analysis identified significant temporal shifts from early TLR, NF-κB, and TNF signaling toward cell-cycle regulation and DNA replication at later stages (FDR < 0.05). Metabolomic analysis identified 721, 1505, 1270, and 849 differentially abundant metabolites (DAMs), respectively, with prominent alterations in lipid, amino acid, and redox-related metabolism. Integrated transcriptomic–metabolomic analysis identified significant stage-specific gene–metabolite associations (|r| > 0.8, *p* < 0.05), including strong associations of (S)-Leucic acid with SAA3 and SGSH and of N-acetyl-L-aspartic acid with TANC1. A dynamic pattern of glutathione depletion, together with disruption of sphingolipid metabolism and enrichment of ferroptosis-related pathways, provided convergent correlative evidence of increasing redox and lipid metabolic stress during infection. Programmed cell death-related pathway analysis showed increasing prominence of apoptosis and cellular senescence at later stages. The RNA-seq findings were further supported by qRT-PCR validation, with strong concordance at all four time points (R = 0.99, *p* ≤ 1.62 × 10^−4^). Collectively, these findings support a time-dependent transition from inflammatory activation to metabolic reprogramming, redox imbalance, and programmed cell death, and provide correlative evidence that ferroptosis may contribute to injury of bovine mammary epithelial cells during *M. bovis* infection.

## 1. Introduction

*Mycoplasmopsis bovis* (*M. bovis*) is a major causative agent of bovine mastitis, pneumonia, arthritis, and reproductive disorders, resulting in considerable economic losses to the dairy industry worldwide [1]. *M. bovis*-associated mastitis is characterized by poor therapeutic efficacy, prolonged disease duration, and frequent recurrence [2], largely owing to the pathogen’s distinctive biological characteristics. These include the absence of a cell wall, which renders many conventional antibiotics ineffective [3], a high genomic AT content associated with genetic variability [4], and sophisticated mechanisms of intracellular survival and immune evasion [5]. Previous studies have demonstrated that *M. bovis* can modulate its adhesion to host cells and evade host immune recognition through the expression of variable surface lipoproteins (Vsps) [6]. In addition, it impairs neutrophil phagocytosis, interferes with macrophage activation, and induces immune cell dysfunction, thereby promoting persistent infection [7,8,9]. Furthermore, *M. bovis* infection has been shown to activate multiple host inflammatory signaling pathways, including nuclear factor-κB (NF-κB), mitogen-activated protein kinase (MAPK), and Toll-like receptor (TLR) signaling, leading to sustained production of pro-inflammatory cytokines such as interleukin (IL)-1β, IL-6, and tumor necrosis factor-α (TNF-α). These responses amplify inflammation and promote tissue damage, which are recognized as key pathogenic events during *M. bovis* infection [10,11,12,13]. Despite substantial progress in understanding the immune responses and bacterial virulence factors associated with *M. bovis* infection, how this pathogen induces metabolic reprogramming and subsequently damages mammary epithelial cells remains unclear. In particular, current evidence regarding energy metabolism [14,15], lipid remodeling [16,17], and redox homeostasis [18] remains fragmented and lacks systematic integration. Therefore, a multi-omics approach is needed to elucidate the pathogenic mechanisms underlying *M. bovis* infection.

Recent studies have shown that immunometabolic reprogramming is an early and rapidly activated response to pathogen infection. By reshaping glycolysis, the tricarboxylic acid (TCA) cycle, and fatty acid and amino acid metabolism, this process profoundly influences host inflammatory responses, redox homeostasis, and cell fate determination [19,20,21]. Pathogen infection frequently induces a metabolic shift resembling the Warburg effect, driving host cells to rely more heavily on aerobic glycolysis for rapid energy production while simultaneously enhancing lipid biosynthesis and remodeling membrane lipid composition to meet the increased demands of inflammatory signaling and cellular structural remodeling [22]. However, although metabolic reprogramming can provide metabolic and structural support for host responses, it may also impair the production of key antioxidant molecules, such as nicotinamide adenine dinucleotide phosphate (NADPH) and glutathione (GSH), thereby promoting excessive accumulation of reactive oxygen species (ROS) and lipid peroxidation, accelerating cellular injury [23,24]. In this context, multiple forms of programmed cell death (PCD), including apoptosis, pyroptosis, and ferroptosis, may serve as important molecular links between metabolic dysregulation, oxidative stress, and tissue damage [25,26,27]. Although accumulating evidence suggests that bacterial infections can induce multiple forms of PCD [28,29], it remains unclear whether *M. bovis* systematically disrupts host metabolic networks to induce oxidative stress and coordinately activate distinct PCD pathways, as well as how these processes evolve over time and are regulated. In particular, whether metabolic reprogramming, oxidative stress, and multiple PCD pathways constitute a continuous cascade that collectively promotes mammary epithelial cell injury and mastitis progression remains to be systematically elucidated.

In the investigation of complex infectious diseases, a single-omics approach is often insufficient to comprehensively elucidate the dynamic regulatory networks underlying host–pathogen interactions. Transcriptomics provides a systematic overview of gene expression patterns and signaling pathway alterations, whereas metabolomics directly reflects cellular metabolic states and functional outputs. These approaches are therefore highly complementary at both the temporal and biological levels. In recent years, the integration of transcriptomics and metabolomics has emerged as an effective strategy for elucidating the sequential cascade from gene regulation to metabolic responses and ultimately to phenotypic alterations, and has been widely applied in studies of infectious diseases, inflammatory responses, and cell fate regulation. Previous studies have demonstrated that integrated multi-omics analyses, particularly the combination of transcriptomics and metabolomics, can systematically characterize host metabolic and immune reprogramming during infection. For example, this strategy has revealed links among energy metabolism, lipid metabolism, and immune signaling in models of *Anaplasma phagocytophilum* infection [30], Japanese encephalitis virus infection [31], and various bacterial infections [32]. In addition, integrated omics approaches have been used to investigate pathogen metabolic adaptation [33], and construct metabolic interaction networks between pathogens and host immune cells [34]. Therefore, the combined analysis of transcriptomics and metabolomics represents a rational approach for systematically elucidating the regulatory mechanisms underlying metabolic disturbances, oxidative stress, and programmed cell death in complex infectious diseases. Based on this rationale, the present study established a time-course model of *M. bovis* PG45 infection in bovine mammary epithelial cells (MAC-T cells) and integrated transcriptomic and untargeted metabolomic analyses to systematically characterize the dynamic interplay among metabolic reprogramming, oxidative stress, and programmed cell death during *M. bovis* infection. This study addresses an existing knowledge gap regarding the metabolic regulation and cellular injury mechanisms underlying *M. bovis*-induced mastitis and provides a theoretical foundation for the development of therapeutic strategies targeting the metabolism–oxidative stress–cell death axis.

## 2. Materials and Methods

### 2.1. Cell Culture and M. bovis Infection Model

An in vitro infection model was established using MAC-T cells (preserved and provided by the Laboratory of Veterinary Microbiology, Gansu Agricultural University) and M. bovis PG45. MAC-T cells were cultured to an appropriate density in 6-well plates before infection. Cells in the infected groups were exposed to *M. bovis* PG45 at a multiplicity of infection (MOI) of 1000 for 4 h. After infection, the culture medium was removed and replaced with fresh medium supplemented with gentamicin at a final concentration of 400 μg/mL, followed by a further 4-h incubation [35]. After removal of the culture medium, the cells were rinsed twice with PBS and then cultured in fresh complete medium. The negative control (NC) group was not infected with *M. bovis*, whereas all other experimental procedures were identical to those used for the infected groups.

### 2.2. Sample Collection

#### 2.2.1. Sample Preparation for Transcriptome Sequencing

For transcriptomic analysis, One NC group and four infected groups were included, each with three biological replicates [36]. Samples were collected at 8, 24, 48, and 72 h post-infection. For sample collection, the medium was removed from each well, and 1 mL of pre-chilled TRIzol reagent (Thermo Fisher Scientific, Waltham, MA, USA) was subsequently added. The samples were repeatedly pipetted until they were completely lysed and detached, and the lysates were subsequently placed in cryogenic tubes and kept at −80 °C until RNA extraction and transcriptomic sequencing.

#### 2.2.2. Metabolomics Sample Preparation

The experimental design was identical to that used for the transcriptomic analysis, except that each group comprised six biological replicates [36]. During sample collection, the cells were detached by trypsin digestion, and digestion was terminated by adding DMEM supplemented with 2% fetal bovine serum (FBS). The cells were subsequently counted, and the corresponding cell numbers were recorded. The collected cell samples were rapidly frozen in liquid nitrogen and maintained at −80 °C until subsequent metabolite extraction and untargeted metabolomic profiling.

### 2.3. Transcriptomic Analysis

Total RNA was isolated from MAC-T cells using standard procedures and subjected to rigorous quality control. Before library preparation, the integrity of the RNA samples was examined with an Agilent 2100 Bioanalyzer (Agilent Technologies, Santa Clara, CA, USA) to ensure their suitability for subsequent library construction. Messenger RNA (mRNA) was then enriched from total RNA using Oligo(dT) magnetic beads (Thermo Fisher Scientific, Waltham, MA, USA). Following mRNA fragmentation, random hexamer primers were utilized for the synthesis of first- and second-strand cDNA. The resulting cDNA fragments subsequently underwent end repair, A-tailing, adapter ligation, fragment selection, PCR amplification, and purification to generate sequencing libraries. Library concentrations were determined using a Qubit fluorometer (Thermo Fisher Scientific, Waltham, MA, USA) and quantitative real-time PCR (Applied Biosystems, Waltham, MA, USA), while fragment-size distributions were evaluated with a Bioanalyzer. Qualified libraries were pooled at appropriate effective concentrations and sequenced using a NovaSeq 6000 platform (Illumina, San Diego, CA, USA).

Quality control of the raw sequencing data was performed with fastp (v0.23.4), including the removal of low-quality reads, adapter sequences, and reads containing ambiguous bases (N). The quality of the processed sequencing data was assessed based on the Q20, Q30, and GC content values. A publicly available database was used to obtain the reference genome and its corresponding annotation file. The reference genome was indexed using HISAT2 (v2.2.1), followed by alignment of the clean reads to the indexed genome. Differentially expressed genes (DEGs) were identified by pairwise comparisons between each infected group and the NC group using DESeq2 (v1.42.0). Genes with adjusted *p* values (padj) ≤ 0.05 and |log_2_FoldChange| ≥ 1 were considered significantly differentially expressed. The false discovery rate (FDR) was used to account for multiple testing, and padj values were used to assess statistical significance after multiple-testing correction. Volcano plots were used to visualize the distribution and significance of differentially expressed genes. The DEGs were subjected to Gene Ontology (GO) and Kyoto Encyclopedia of Genes and Genomes (KEGG) enrichment analyses using topGO (v4.1.0) and clusterProfiler (v3.8.1), respectively. Protein–protein interaction (PPI) information was retrieved from the STRING database (v11.5), followed by construction and visualization of the PPI network in NetworkAnalyst 3.0 (https://www.networkanalyst.ca/).

### 2.4. Metabolomics Analysis

Cell samples were transferred to Eppendorf tubes (Eppendorf SE, Hamburg, Germany), followed by the addition of 300 μL of 80% methanol to each sample. Following 5 min of snap-freezing in liquid nitrogen, the samples were thawed on ice, vortexed for 30 s, and sonicated for 6 min, followed by centrifugation at 5000 rpm and 4 °C for 1 min. The resulting supernatants were collected in fresh tubes and subsequently lyophilized to obtain dry extracts. The dried extracts were reconstituted in 10% methanol to the original sample volume and subjected to liquid chromatography–mass spectrometry (LC-MS) analysis. A Vanquish UHPLC system (Thermo Fisher Scientific, Waltham, MA, USA) coupled with a Q Exactive HF-X mass spectrometer (Thermo Fisher Scientific, Darmstadt, Germany) was employed for metabolomic profiling. Samples were chromatographically separated on a Hypersil Gold column (100 × 2.1 mm, 1.9 μm; Thermo Fisher Scientific, Waltham, MA, USA), with the column temperature maintained at 40 °C and the flow rate set to 0.2 mL/min. Mass spectrometric detection was performed in alternating positive- and negative-ion electrospray ionization (ESI) modes. The spray voltage, capillary temperature, sheath gas flow rate, and auxiliary gas flow rate were set to 3.2 kV, 320 °C, 40 arbitrary units (arb), and 10 arb, respectively. The mass range was set to *m*/*z* 100–1500, and data-dependent MS/MS acquisition was used for secondary fragment analysis. Raw data were subjected to peak alignment in the database using retention time (±0.2 min) and mass-to-charge ratio (±5 ppm) as matching criteria. Peak extraction and quantification were performed according to predefined criteria for mass accuracy, signal-intensity variation (±30%), and signal-to-noise ratio (S/N) ≥ 3. Molecular formulas were deduced and matched against databases such as mzCloud (https://www.mzcloud.org). Blank controls were used to eliminate interfering signals, followed by data normalization to ensure consistency. Data processing was performed in a Linux (CentOS 6.6) environment using R (v3.4.3) and Python (v3.5.0). The KEGG (https://www.genome.jp/kegg/pathway.html, accessed on 20 October 2025), Human Metabolome Database (HMDB) (https://hmdb.ca/metabolites, accessed on 20 October 2025), and LIPID MAPS (http://www.lipidmaps.org/, accessed on 20 October 2025) were searched for metabolite annotation. Principal component analysis (PCA) and partial least squares discriminant analysis (PLS-DA) were subsequently conducted in metaX (v2.1.1). PLS-DA is a supervised multivariate statistical method used to discriminate samples according to their metabolic profiles, and variable importance in projection (VIP) scores were derived to evaluate the contribution of individual metabolites to group discrimination. Student’s *t*-test was used for *p* value calculation, and fold changes (FCs) were subsequently calculated. Differentially abundant metabolites (DAMs) were identified by pairwise comparisons between each infected group and the NC group based on VIP scores, FCs, and *p* values. Metabolites with VIP > 1.0, FC > 1.2 or FC < 0.833, and *p* < 0.05 were considered significantly differentially abundant. The R package ggplot2 (v3.5.1) was employed to generate bubble plots. For metabolic pathway enrichment, x/n > y/N was used as the enrichment criterion, and pathways with *p* < 0.05 were considered significantly enriched.

### 2.5. Integrated Transcriptomic and Metabolomic Analysis

The associations between DEGs and DAMs were evaluated by calculating Pearson correlation coefficients. KEGG pathway mapping was performed for the DEGs and DAMs to identify common pathways between the transcriptomic and metabolomic datasets, followed by an integrated evaluation of the key biochemical, metabolic, and signal transduction pathways. Gene–metabolite interaction networks were constructed and visualized using igraph (v1.6.0).

### 2.6. Quantitative Real-Time PCR (qRT-PCR)

Bovine programmed cell death-related genes were obtained from the database (https://amigo.geneontology.org/amigo/term/GO:0012501, accessed on 20 October 2025) and cross-referenced with genes expressed at each post-infection time point. The overlapping genes identified at each time point were subjected to KEGG pathway analysis, and pathway maps were visualized using the Weishengxin platform (https://www.bioinformatics.com.cn/, accessed on 20 October 2025). At each time point, five overlapping genes showing the most pronounced changes in expression were selected, yielding a total of 20 candidate genes. The genes selected for validation at each time point were as follows: BCL2A1, NFKBIZ, ZC3H12A, TNFAIP3, and GZMK at 8 h; BCL2A1, BMX, CHI3L2, CYP26B1, and ADAMTS20 at 24 h; BMF, TNFAIP3, CDCA7, HELLS, and TOP2A at 48 h; and BMF, CARD14, IL6, TOP2A, and CDCA7 at 72 h. Primer sequences were designed with NCBI Primer-BLAST (https://www.ncbi.nlm.nih.gov/, accessed on 20 October 2025), and their specificity was subsequently verified online. Detailed primer information is provided in Supplementary Table S1. For qRT-PCR validation, three biological replicates were examined at each infection time point using the same biological samples subjected to transcriptomic sequencing. Quantitative real-time PCR (qRT-PCR) was used to quantify transcript levels. cDNA was synthesized using HiScript Q RT SuperMix for qPCR (Vazyme, Nanjing, China) in accordance with the manufacturer’s instructions. The subsequent qPCR reaction was prepared in a final volume of 20 μL, containing 10 μL of ChamQ Universal SYBR qPCR Master Mix (Vazyme, Nanjing, China), 0.5 μL each of forward and reverse primers (Sangon Biotech, Shanghai, China), 7 μL of nuclease-free water, and 2 μL of cDNA template. Amplification was carried out on a LightCycler 480 Real-Time PCR System (Roche Diagnostics, Basel, Switzerland). Each sample was analyzed using three technical replicates, with β-actin used as the reference gene. Relative gene expression levels were calculated using the 2^−ΔΔCt^ method.

## 3. Results

### 3.1. Transcriptional Changes Induced by M. bovis Infection

**Figure 1:**
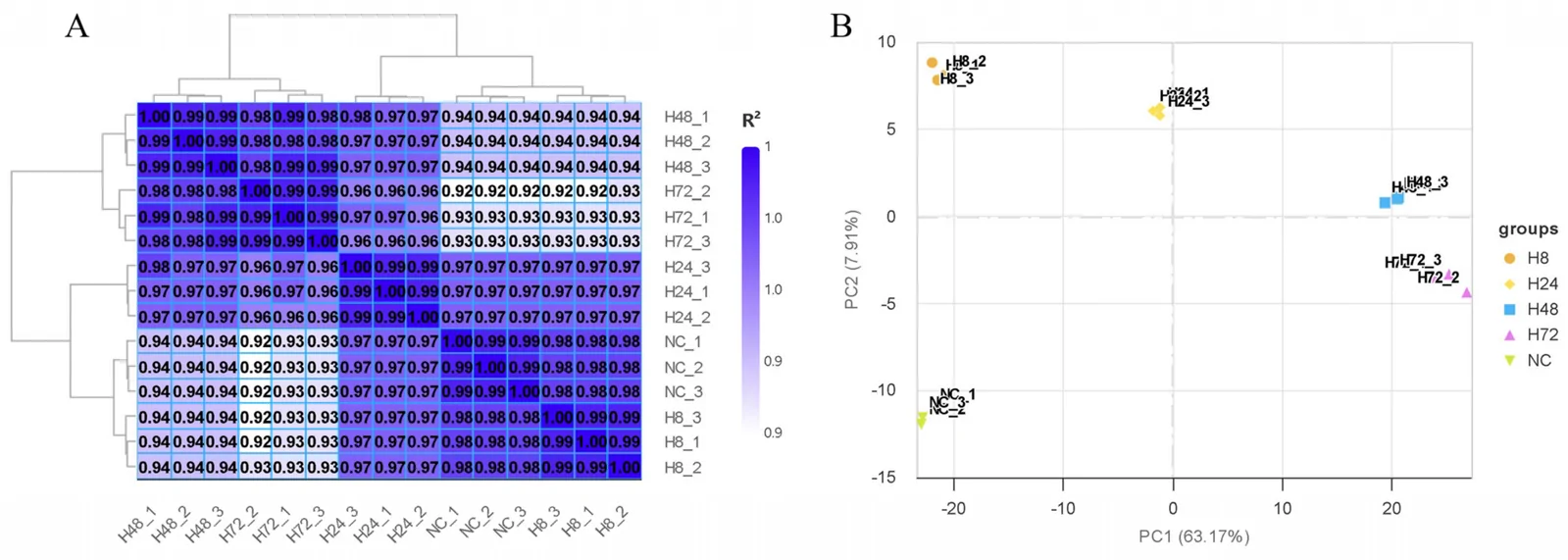
Transcriptomic changes in MAC-T cells following *M. bovis* infection. (**A**) Heatmap showing sample correlations. (**B**) Principal component analysis (PCA) score plot.

**Figure 2:**
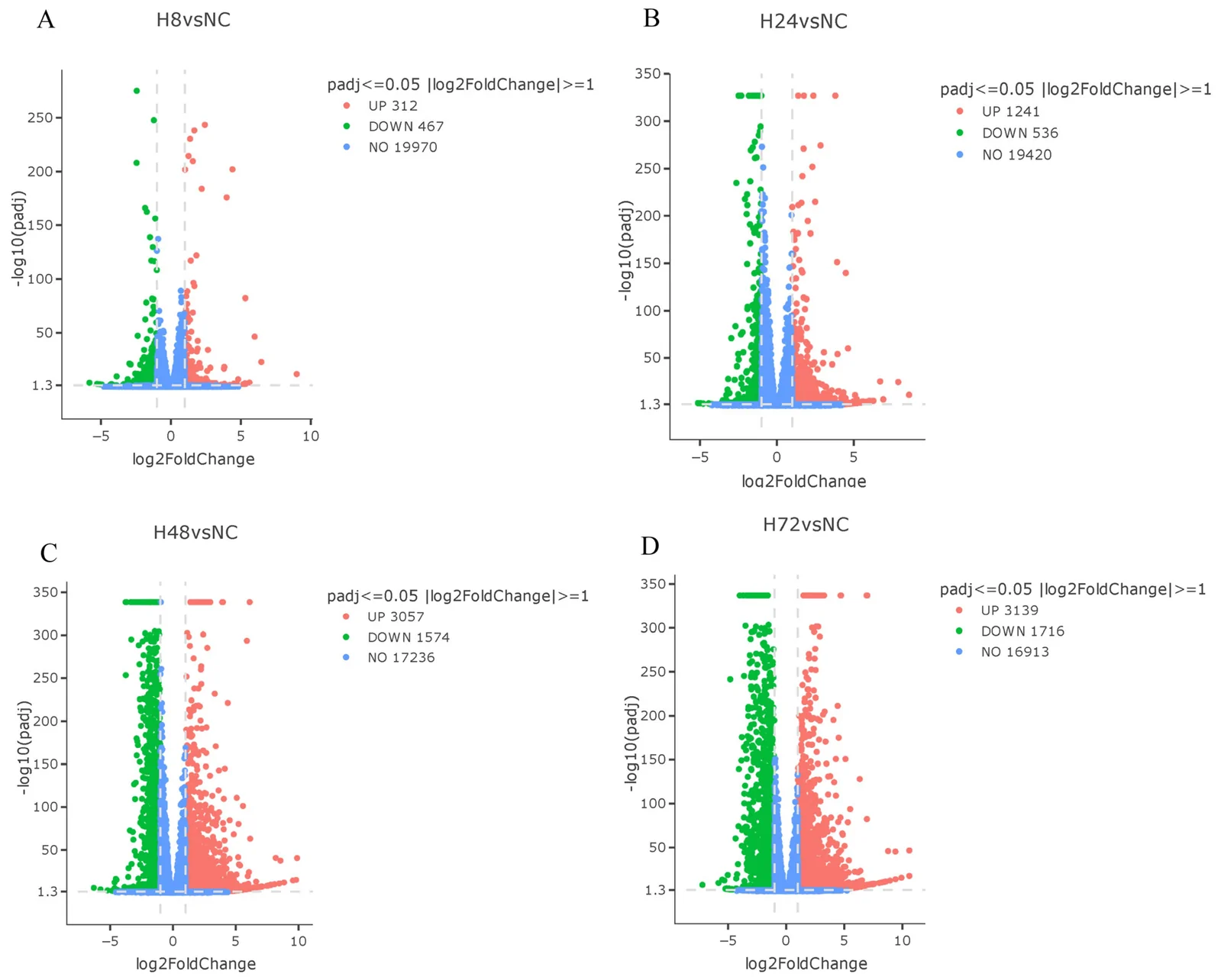
Transcriptomic changes in MAC-T cells following *M. bovis* infection, as shown by volcano plots. (**A**) H8 vs. NC; (**B**) H24 vs. NC; (**C**) H48 vs. NC; (**D**) H72 vs. NC.

Transcriptome sequencing of 15 samples generated 97.15 Gb of clean bases. The number of clean bases obtained for each sample exceeded 6.12 Gb, and the percentages of Q20 and Q30 bases were above 99.23% and 96.94%, respectively. Correlation analysis revealed significant co-expression patterns among genes (*p* < 0.05), which were visualized using a heatmap (Figure 1A). Meanwhile, principal component analysis (PCA) confirmed substantial differences between the groups (Figure 1B). Transcriptome profiling identified 18,919 expressed genes, comprising 18,331 known genes and 588 novel genes. The number of DEGs increased progressively with infection duration, from 779 DEGs in the H8 vs. NC comparison to 1777, 4631 and 4855 DEGs in the H24 vs. NC, H48 vs. NC and H72 vs. NC comparisons, respectively. At 8 h, 312 genes were upregulated and 467 were downregulated; at 24 h, 1241 were upregulated and 536 were downregulated; at 48 h, 3057 were upregulated and 1574 were downregulated; and at 72 h, 3139 were upregulated and 1716 were downregulated (Figure 2).

**Figure 3:**
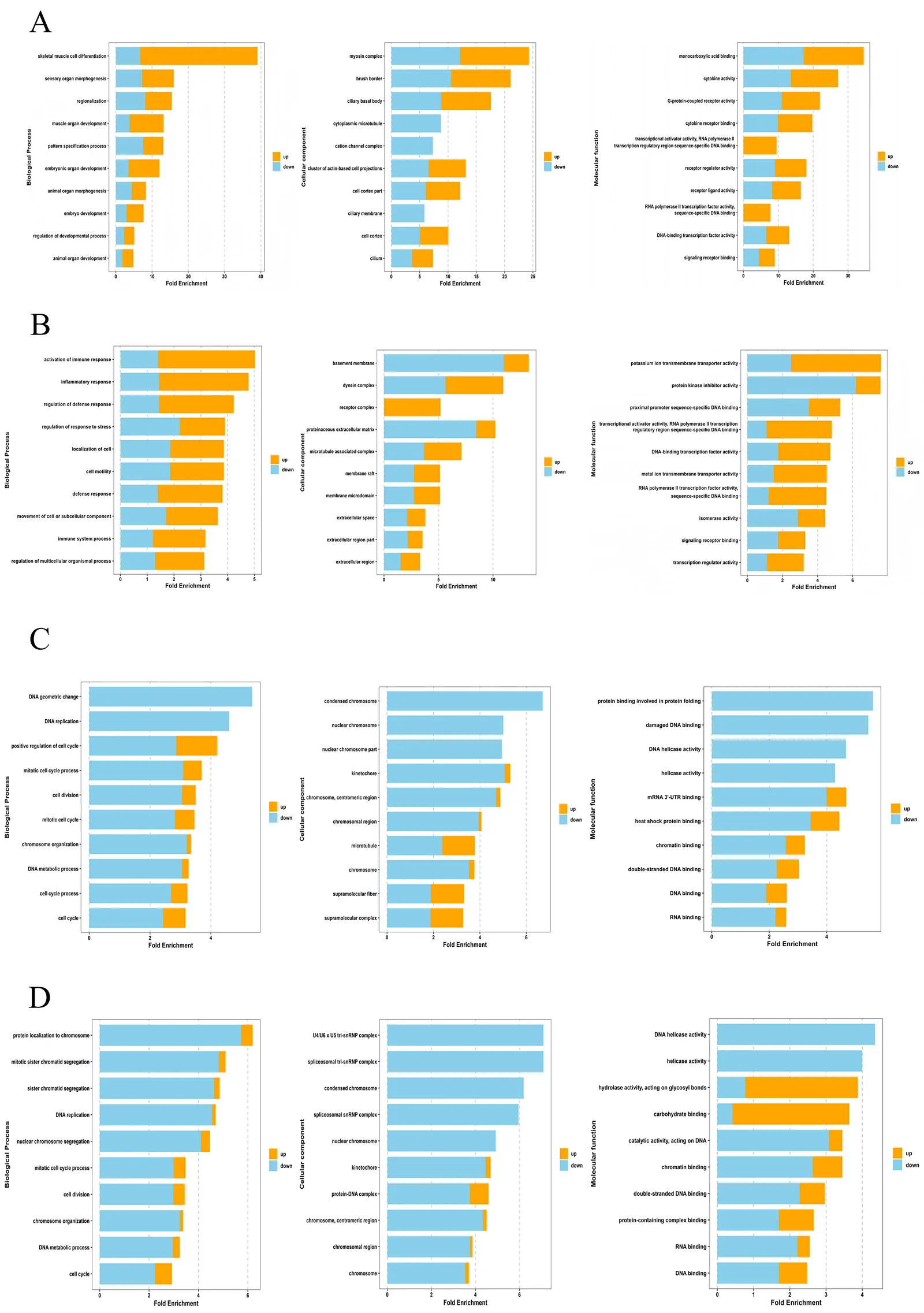
GO enrichment analysis of DEGs in MAC-T cells following *M. bovis* infection. (**A**) H8 vs. NC; (**B**) H24 vs. NC; (**C**) H48 vs. NC; (**D**) H72 vs. NC.

GO enrichment analysis identified 1966, 2709, 3220, and 3207 significantly enriched GO terms (FDR < 0.05) in the H8 vs. NC, H24 vs. NC, H48 vs. NC, and H72 vs. NC comparisons, respectively. These terms comprised biological process (BP), cellular component (CC), and molecular function (MF) categories, with BP accounting for the largest proportion at all time points. The enrichment patterns showed clear temporal changes in the biological functions affected by *M. bovis* infection. At 8 h, upregulated genes were mainly associated with developmental processes, including skeletal muscle cell differentiation, animal organ development, muscle organ development, and striated muscle tissue development, whereas downregulated genes were enriched in lipid metabolic and biosynthetic processes, together with animal organ morphogenesis and pattern specification processes. At 24 h, the functional profile shifted toward a stronger immune and inflammatory response, with upregulated genes enriched in activation of immune response, inflammatory response, defense response, and immune system process. In contrast, downregulated genes were predominantly associated with cell division and cell-cycle-related processes, including cell division, cell cycle process, and cell cycle. At 48 h, the enrichment pattern further emphasized host defense and immune-related functions. Upregulated genes were enriched in system process, cell adhesion, defense response, and immune response, whereas downregulated genes showed prominent enrichment in DNA- and chromosome-related processes, including DNA geometric change, DNA replication, chromosome organization, and DNA metabolic process. A similar pattern persisted at 72 h, with upregulated genes enriched in cell adhesion, regulation of cytokine production, cytokine production, defense response, and immune response, while downregulated genes were mainly associated with chromosome segregation and DNA replication, including protein localization to chromosome, mitotic sister chromatid segregation, sister chromatid segregation, and DNA replication. Overall, the GO enrichment profiles indicated a temporal transition from early metabolic and developmental alterations toward sustained immune and inflammatory activation, accompanied by progressive suppression of cell-cycle and DNA replication-related processes during the later stages of infection (Figure 3).

**Figure 4:**
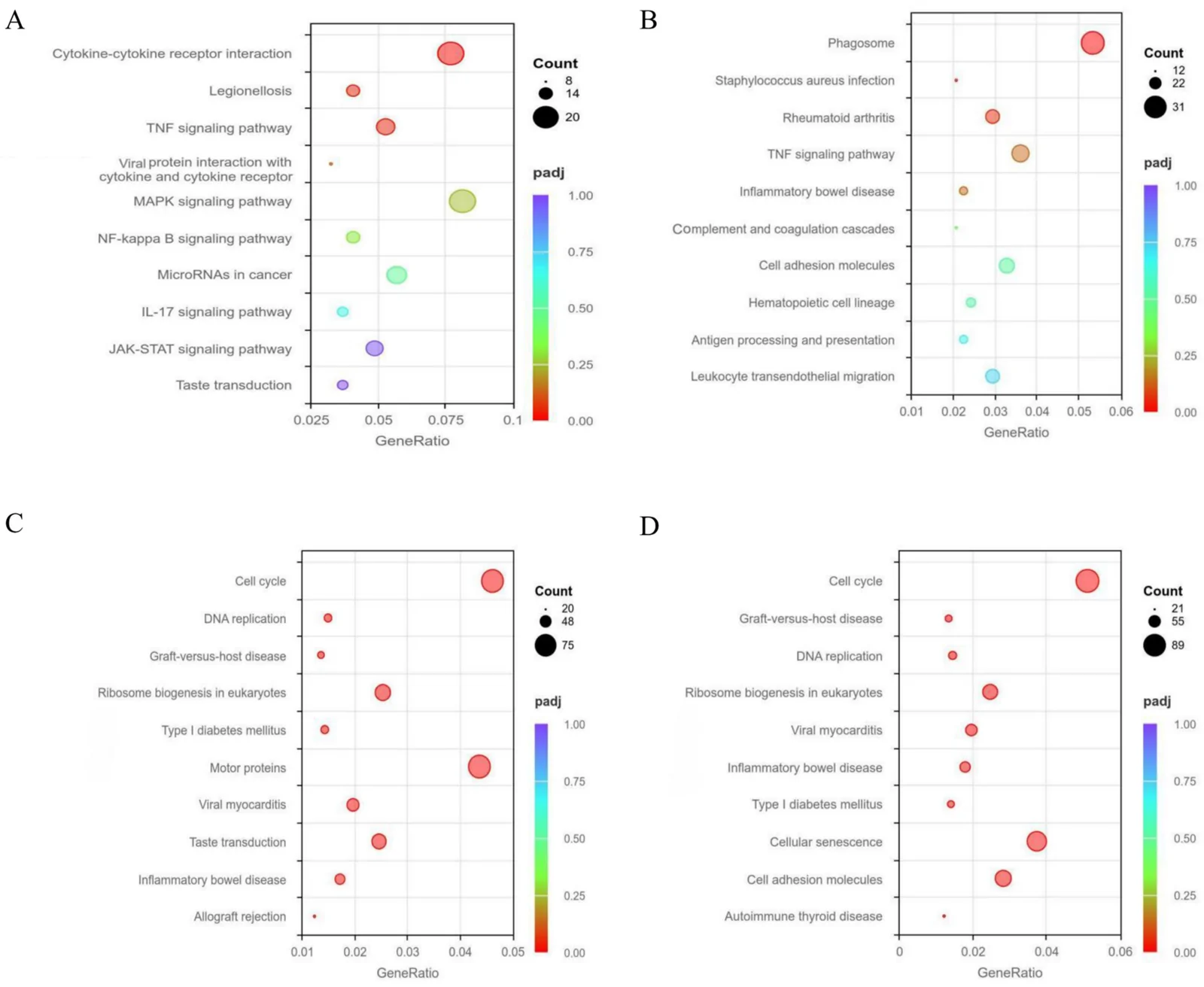
KEGG pathway enrichment of DEGs in MAC-T cells following *M. bovis* infection. (**A**) H8 vs. NC; (**B**) H24 vs. NC; (**C**) H48 vs. NC; (**D**) H72 vs. NC.

KEGG enrichment analysis was performed to characterize the biological pathways associated with the DEGs. A total of 275, 326, 338, and 340 significantly enriched KEGG pathways were identified in the H8 vs. NC, H24 vs. NC, H48 vs. NC, and H72 vs. NC comparisons, respectively (FDR ≤ 0.05). Enriched pathways were ranked according to their GeneRatio to compare the enrichment profiles across infection stages. The pathway enrichment patterns showed a clear temporal shift from early inflammatory and signal-transduction responses toward pathways associated with cellular proliferation and DNA replication at later stages. At 8 h, the top-ranked pathways included Cytokine–cytokine receptor interaction, Legionellosis, and TNF signaling pathways. Upregulated DEGs were mainly enriched in the NF-κB signaling, TNF signaling, Viral protein interaction with cytokine and cytokine receptor, and IL-17 signaling pathways, whereas downregulated DEGs were primarily enriched in the Taste transduction and the MAPK signaling pathways. These results indicated that inflammatory and immune-related signaling pathways were prominent during the early response to *M. bovis* infection. At 24 h, the enrichment profile shifted toward immune- and inflammation-associated pathways, with the Phagosome, *Staphylococcus aureus* infection, Rheumatoid arthritis, and TNF signaling pathways among the top-ranked pathways. These immune- and inflammation-associated pathways were predominantly enriched among the upregulated DEGs. By contrast, downregulated DEGs were mainly associated with Cytoskeleton in muscle cells. These findings suggested sustained activation of host immune and inflammatory responses at the intermediate stage of infection. The pathway profile became more strongly associated with cell-cycle and DNA-related processes at 48 and 72 h. At 48 h, Cell cycle, DNA replication, and Graft-versus-host disease were among the top-ranked pathways. Upregulated DEGs were mainly enriched in Graft-versus-host disease and Type I diabetes mellitus, whereas downregulated DEGs were mainly enriched in Cell cycle, Ribosome biogenesis in eukaryotes, and DNA replication. A similar pattern was observed at 72 h, when Cell cycle, Graft-versus-host disease, and DNA replication were among the top-ranked pathways. Upregulated DEGs were mainly enriched in Graft-versus-host disease, while downregulated DEGs were mainly associated with Cell cycle and Ribosome biogenesis in eukaryotes (Figure 4). Overall, KEGG enrichment analysis revealed a temporal transition from prominent inflammatory and signaling responses at the early stage of infection to increased enrichment of cell-cycle, ribosome biogenesis, and DNA replication-related pathways at the later stages, particularly among the downregulated DEGs.

### 3.2. Metabolic Alterations Induced by M. bovis Infection

**Figure 5:**
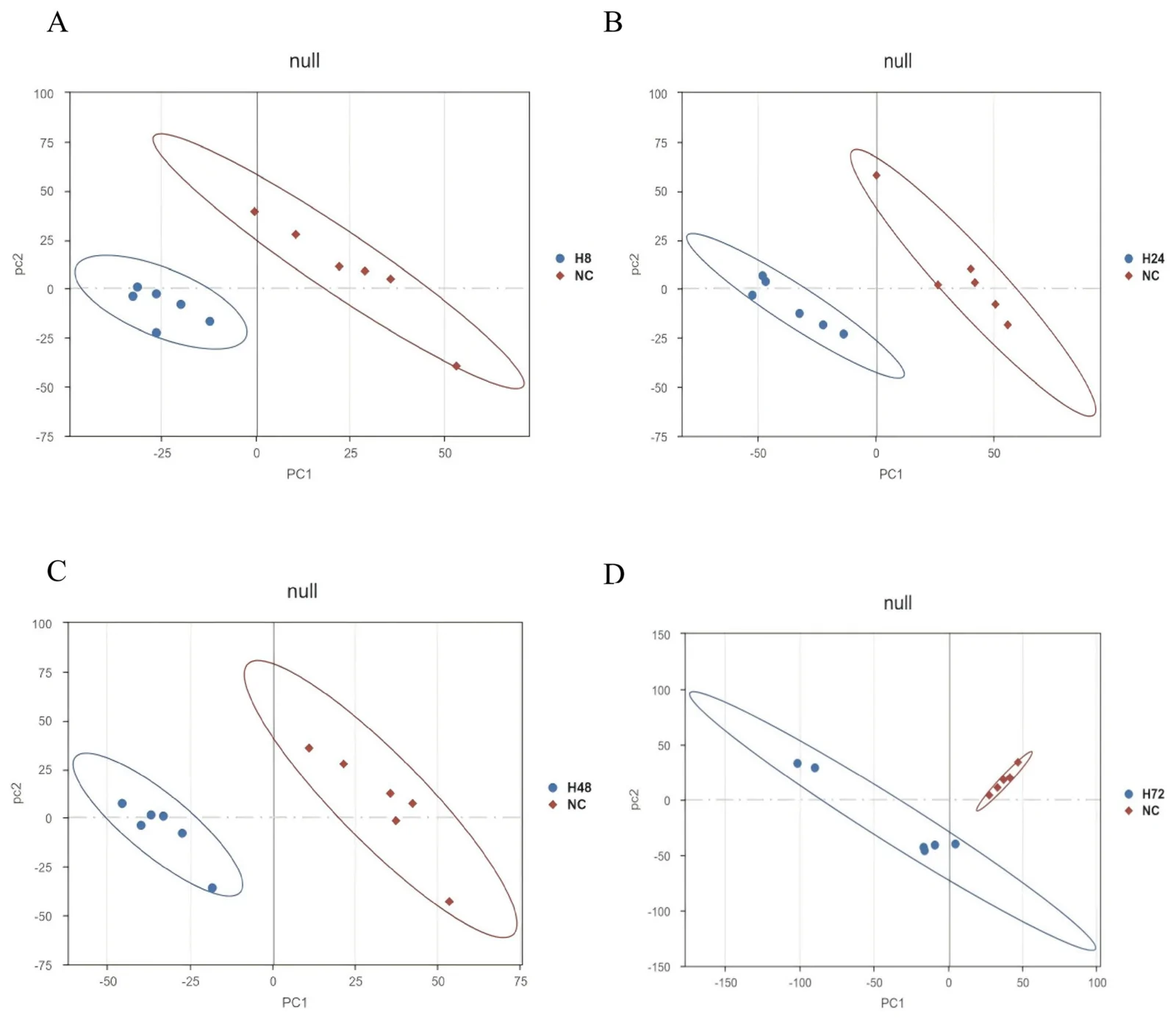
Partial least squares discriminant analysis (PLS-DA) score plots based on the combined positive- and negative-ion metabolomic data from MAC-T cells following *M. bovis* infection. (**A**) H8 vs. NC; (**B**) H24 vs. NC; (**C**) H48 vs. NC; (**D**) H72 vs. NC.

An untargeted metabolomic approach was adopted to characterize the metabolic signatures of MAC-T cells infected with *M. bovis* PG45 and uninfected control cells. Data obtained in positive and negative ionization modes were combined for subsequent analyses. Multivariate statistical analysis showed clear separation between the infected groups (H8, H24, H48, and H72) and the NC group, while replicates from the same group tended to cluster together (Figure 5). These results suggested that *M. bovis* infection was associated with distinct metabolic profiles across the examined time points and demonstrated good consistency among biological replicates, providing a solid basis for subsequent identification of differential metabolites and pathway alterations.

**Figure 6:**
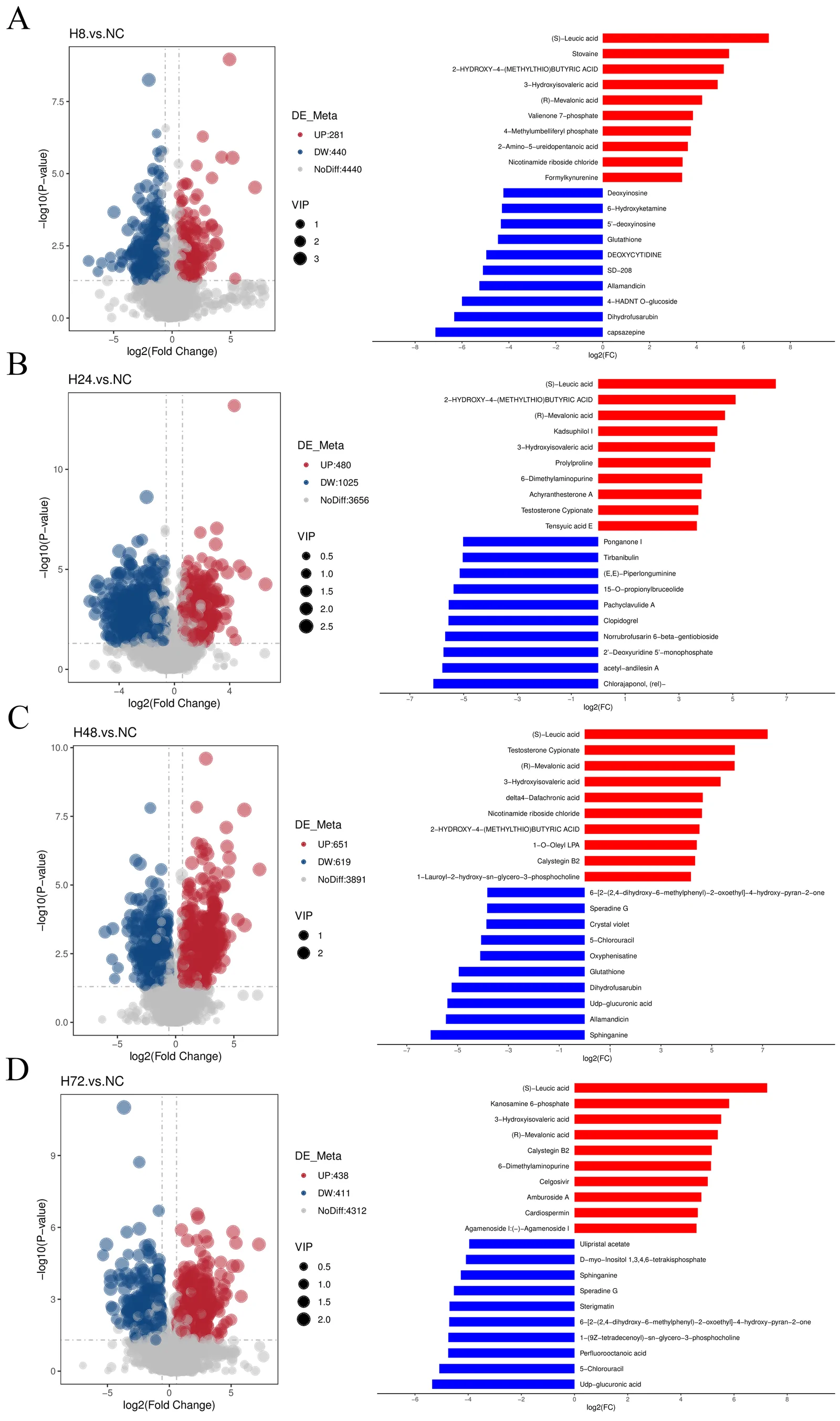
Metabolomic alterations in MAC-T cells following *M. bovis* infection. Volcano plots and the top 20 DAMs based on the combined positive- and negative-ion metabolomic data. (**A**) H8 vs. NC; (**B**) H24 vs. NC; (**C**) H48 vs. NC; (**D**) H72 vs. NC. Red and blue indicate the top 10 upregulated and top 10 downregulated metabolites, respectively.

Based on OPLS-DA VIP scores, statistical significance (*p* < 0.05), and FC, DAMs were identified for the H8 vs. NC, H24 vs. NC, H48 vs. NC, and H72 vs. NC comparisons. The number of DAMs varied substantially across infection stages, increasing from 721 at 8 h to a maximum of 1505 at 24 h, followed by 1270 at 48 h and 849 at 72 h. The proportions of upregulated and downregulated metabolites also changed over time, with downregulated metabolites predominating at 8 and 24 h, whereas the numbers of upregulated and downregulated metabolites became more comparable at 48 and 72 h. The differential metabolite profiles further revealed several metabolites that changed markedly across multiple infection stages. Notably, (S)-Leucic acid was consistently among the most strongly upregulated metabolites at all four time points, whereas UDP-glucuronic acid showed marked downregulation at both 48 and 72 h. Other highly altered metabolites displayed more time-specific patterns. At 8 h, the most strongly upregulated metabolites included (S)-Leucic acid, Stovaine, and 2-Hydroxy-4-(methylthio)butyric acid, while capsazepine, Dihydrofusarubin, and 4-HADNT O-glucoside were among the most strongly downregulated metabolites. At 24 h, (S)-Leucic acid, 2-Hydroxy-4-(methylthio)butyric acid, and (R)-Mevalonic acid showed the largest increases, whereas Chlorajaponol, (rel)-, acetyl-andilesin A, and 2’-Deoxyuridine 5’-monophosphate showed the largest decreases. At 48 h, (S)-Leucic acid, Testosterone Cypionate, and (R)-Mevalonic acid were the three most upregulated metabolites, whereas Sphinganine, Allamandicin, and UDP-glucuronic acid were the three most downregulated metabolites. At 72 h, the most strongly upregulated metabolites were (S)-Leucic acid, Kanosamine 6-phosphate, and 3-Hydroxyisovaleric acid, while UDP-glucuronic acid, 5-Chlorouracil, and Perfluorooctanoic acid showed the greatest decreases (Figure 6).

**Figure 7:**
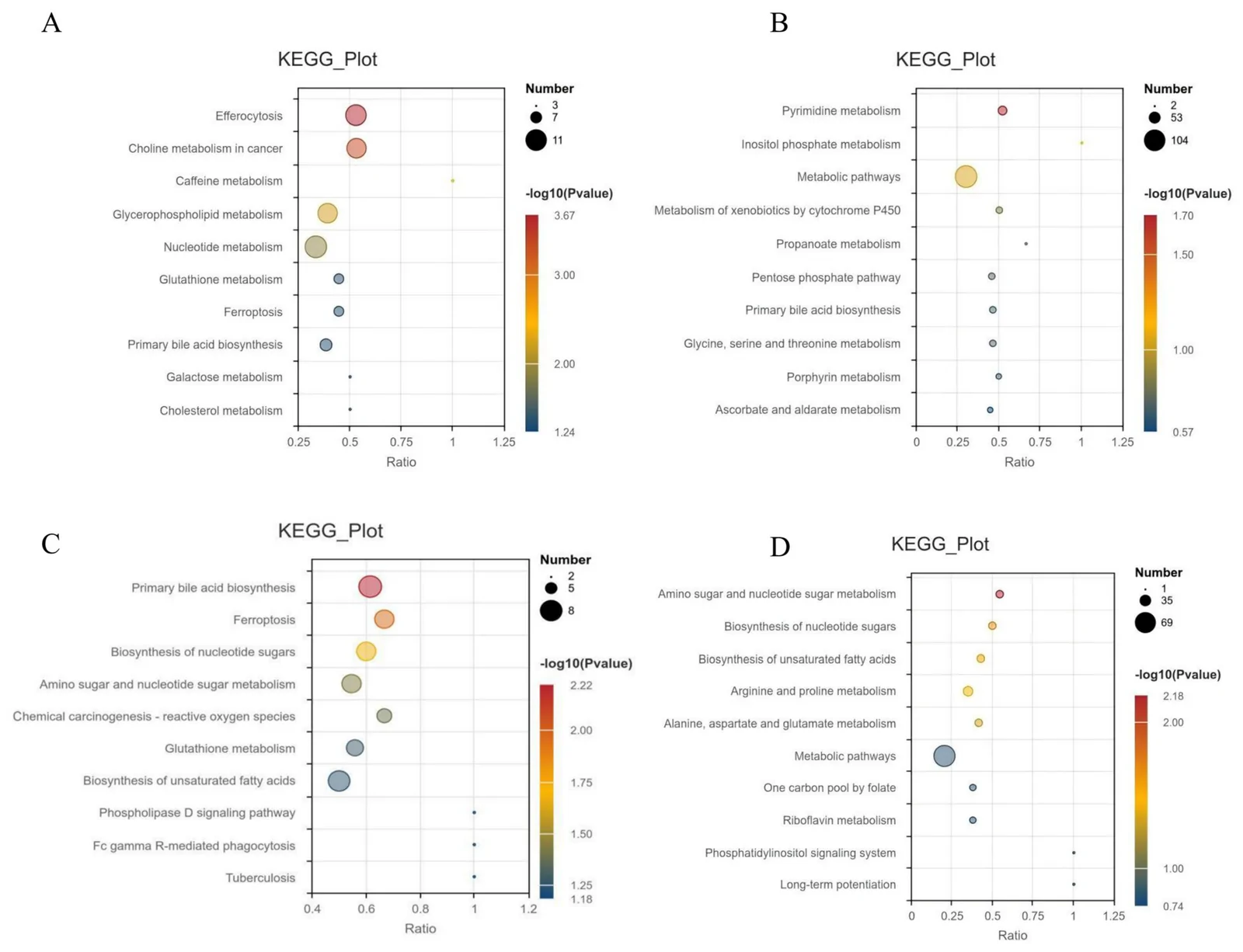
KEGG pathway enrichment analysis of DAMs in MAC-T cells following *M. bovis* infection. (**A**) H8 vs. NC; (**B**) H24 vs. NC; (**C**) H48 vs. NC; (**D**) H72 vs. NC.

KEGG enrichment analysis was conducted on stage-specific DAMs to identify the metabolic pathways involved at each time point. The top 10 enriched pathways were selected for visualization using bubble plots, revealing distinct and time-dependent metabolic enrichment profiles following *M. bovis* infection (Figure 7). At 8 h, the enriched pathways included Efferocytosis, Choline metabolism in cancer, Glycerophospholipid metabolism, Nucleotide metabolism, Glutathione metabolism, and Ferroptosis, indicating early alterations involving lipid, nucleotide, and redox-related metabolic processes. At 24 h, the enrichment profile was characterized predominantly by Pyrimidine metabolism, Inositol phosphate metabolism, Metabolic pathways, Pentose phosphate pathway, and Primary bile acid biosynthesis, suggesting broader remodeling of nucleotide and intermediary metabolism. At 48 h, several lipid- and redox-associated pathways became prominent, including Primary bile acid biosynthesis, Ferroptosis, Glutathione metabolism, Biosynthesis of unsaturated fatty acids, and Chemical carcinogenesis–reactive oxygen species. By 72 h, the enrichment profile shifted toward Amino sugar and nucleotide sugar metabolism, Biosynthesis of nucleotide sugars, Biosynthesis of unsaturated fatty acids, Arginine and proline metabolism, and Alanine, aspartate and glutamate metabolism. Collectively, these results indicate that metabolic alterations during *M. bovis* infection were dynamically reorganized across infection stages, with lipid- and redox-associated pathways evident during both early and later stages and nucleotide- and amino acid-related pathways becoming prominent at different stages of infection.

### 3.3. Transcriptome–Metabolome Correlation Analysis

**Figure 8:**
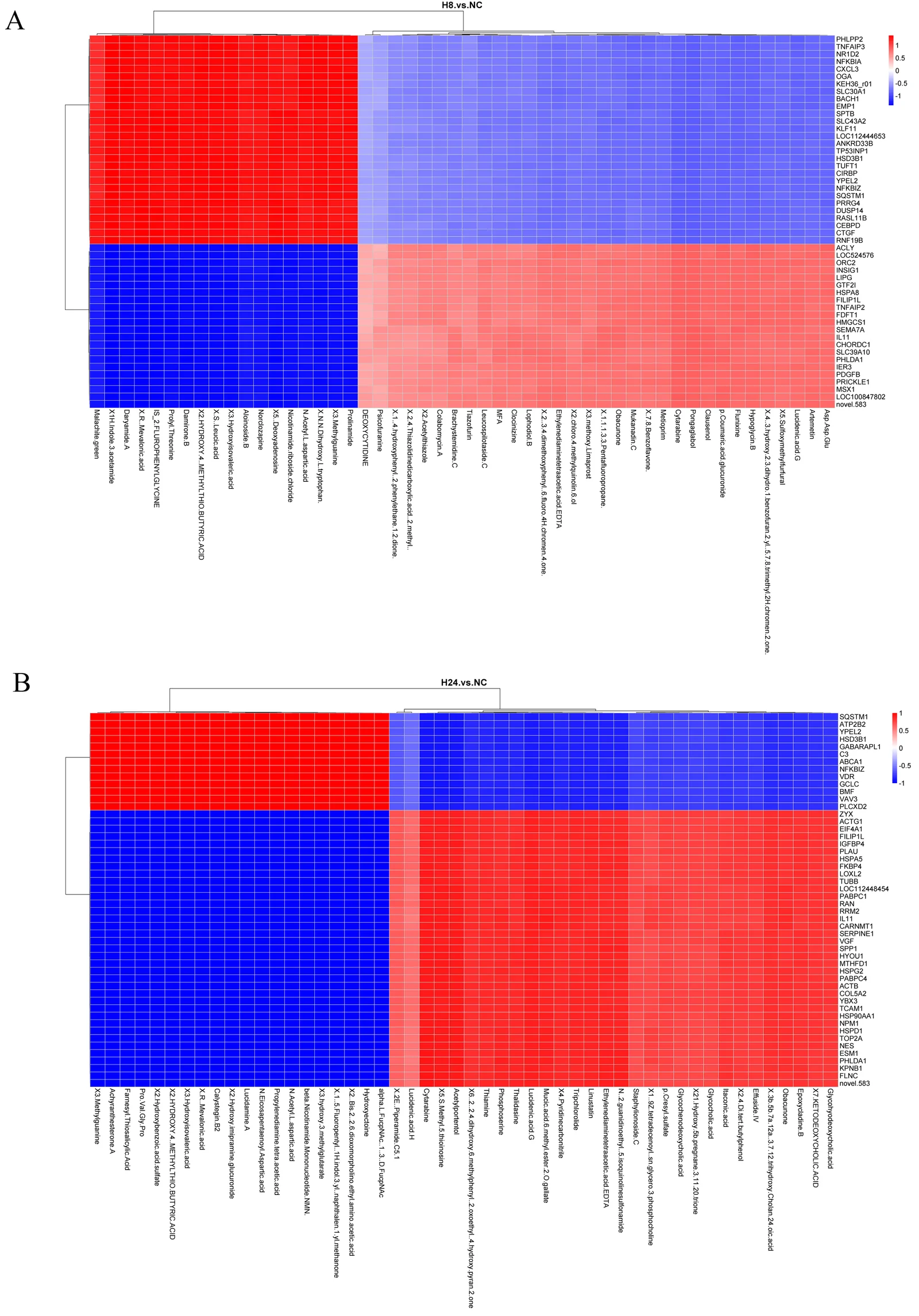

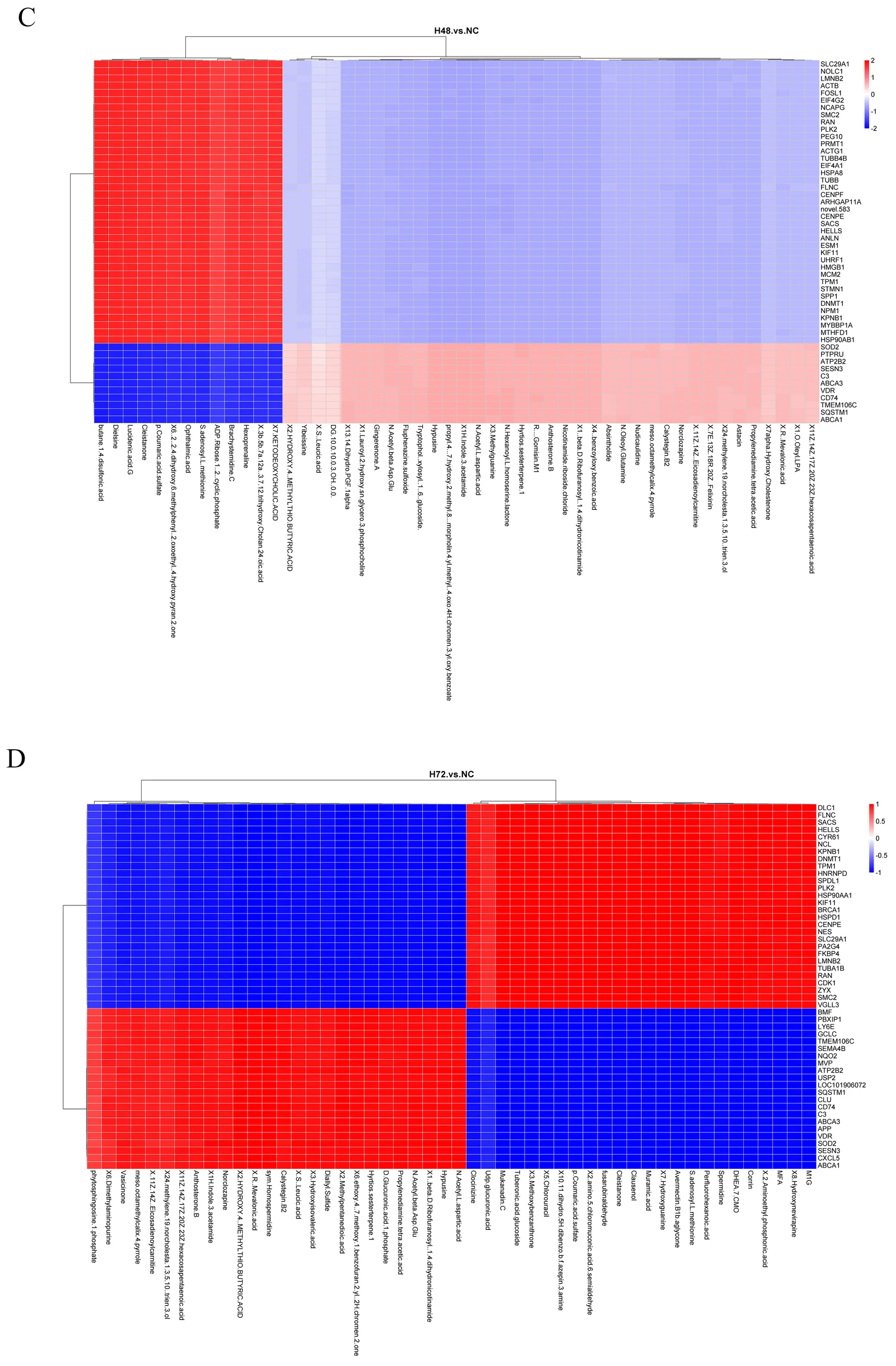
Integrated correlation analysis of transcriptomic and metabolomic profiles in MAC-T cells following *M. bovis* infection. Correlation heatmaps of the top 50 DEGs and DAMs. (**A**) H8 vs. NC; (**B**) H24 vs. NC; (**C**) H48 vs. NC; (**D**) H72 vs. NC.

**Figure 9:**
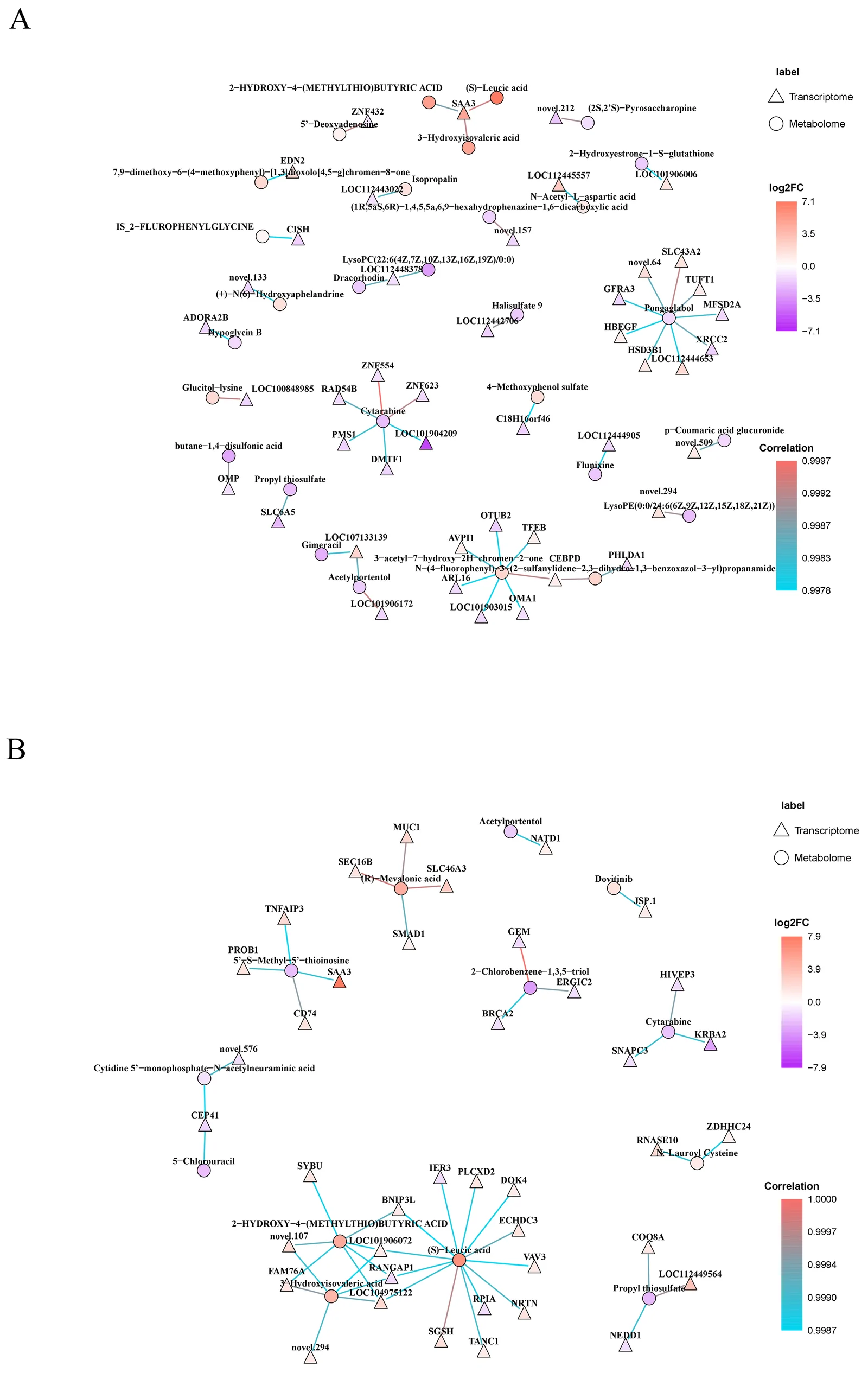

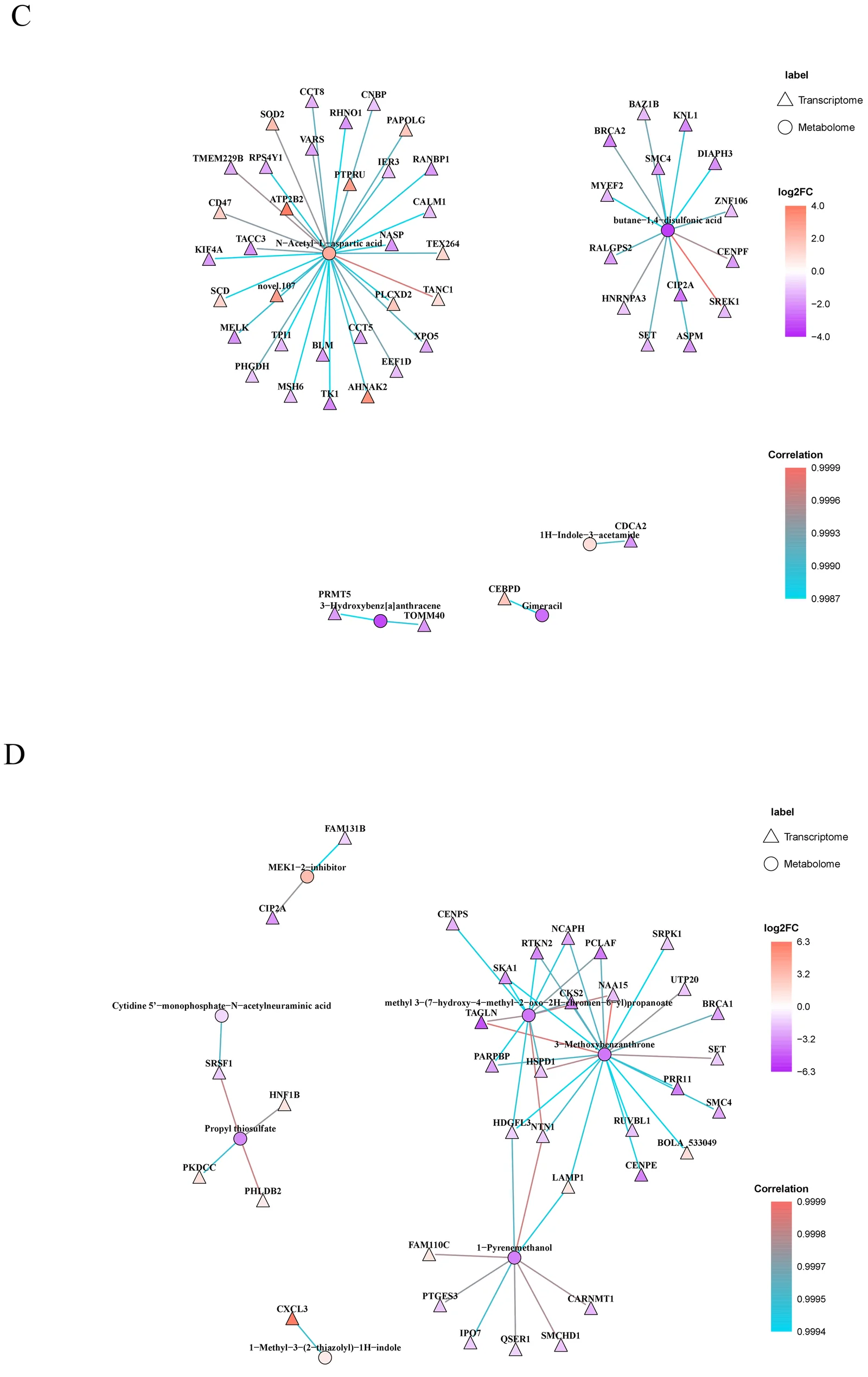
Integrated correlation analysis of transcriptomic and metabolomic profiles in MAC-T cells following *M. bovis* infection. Gene–metabolite interaction networks of DEGs and DAMs. (**A**) H8 vs. NC; (**B**) H24 vs. NC; (**C**) H48 vs. NC; (**D**) H72 vs. NC.

Multi-omics correlation analysis revealed associations between transcriptomic and metabolomic alterations during *M. bovis* PG45 infection. Pearson correlation coefficients were calculated between significantly DEGs and DAMs, and gene–metabolite pairs with |r| > 0.8 and *p* < 0.05 were considered significantly correlated. The correlation patterns varied across infection stages, with distinct clustering structures observed in the heatmaps of the top 50 DAMs and their correlated DEGs (Figure 8). At 8 h post-infection, the correlation pattern was relatively focused, with several metabolites showing strong associations with inflammation-related genes. Among these, SAA3 exhibited a strong correlation with (S)-Leucic acid. At 24 h, the correlation network became more extensive, and SGSH showed a strong association with (S)-Leucic acid. At 48 h, a more complex gene–metabolite interaction pattern was observed, with multiple highly correlated genes and metabolites forming interconnected clusters. Representative associations included TANC1 with N-acetyl-L-aspartic acid and SREK1 with butane-1,4-disulfonic acid. At 72 h, extensive gene–metabolite correlation patterns were observed, with multiple genes and metabolites forming highly connected interaction clusters. Notably, NAA15 showed a strong association with 3-Methoxybenzanthrone (Figure 9). Overall, the correlation networks displayed distinct stage-dependent patterns, suggesting that the molecular response to *M. bovis* infection involved coordinated changes in gene expression and metabolite abundance over the course of infection.

### 3.4. KEGG Pathway Enrichment and qPCR Validation of Programmed Cell Death-Related Genes

**Figure 10:**
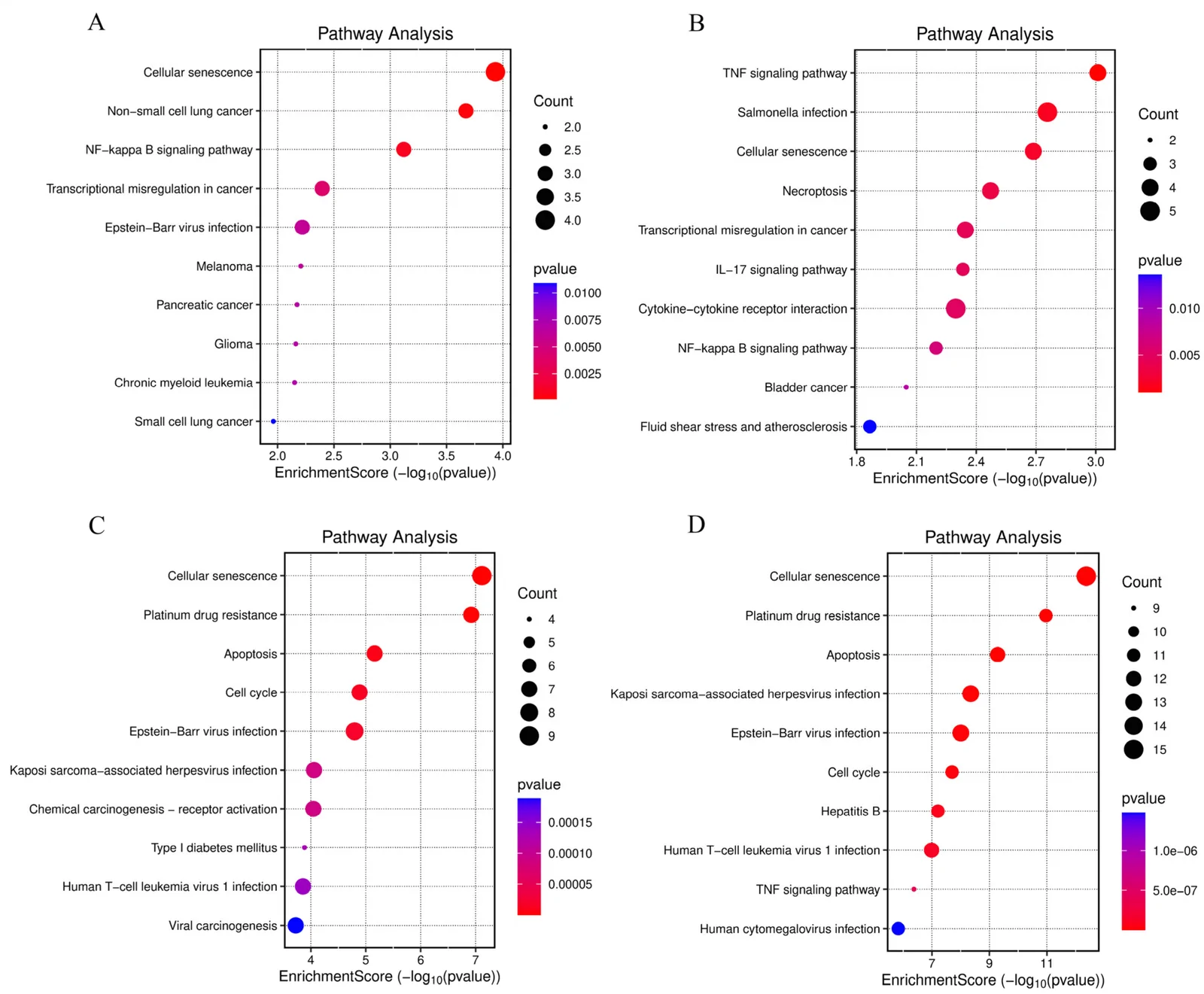
KEGG pathway enrichment analysis of programmed cell death-related genes. (**A**) 8 h; (**B**) 24 h; (**C**) 48 h; (**D**) 72 h.

Genes expressed at each post-infection time point were compared with bovine programmed cell death-related genes, and the overlapping genes identified at each time point were subjected to KEGG enrichment analysis. Bubble plots were used to illustrate the 10 most significantly enriched pathways (Figure 10). The enrichment patterns varied across the infection stages. At 8 h post-infection, the overlapping genes were mainly enriched in the Cellular senescence, Non-small cell lung cancer, and NF-kappa B signaling pathways. At 24 h, enrichment shifted toward the TNF signaling pathway and *Salmonella* infection, while Cellular senescence remained among the major enriched pathways. By 48 h, Cellular senescence, Platinum drug resistance, and Apoptosis emerged as the predominant enriched pathways, and these pathways remained prominent at 72 h. Overall, the enrichment patterns indicated a dynamic change in programmed cell death-related pathways during infection, with Cellular senescence and Apoptosis becoming increasingly prominent at the later stages.

**Figure 11:**
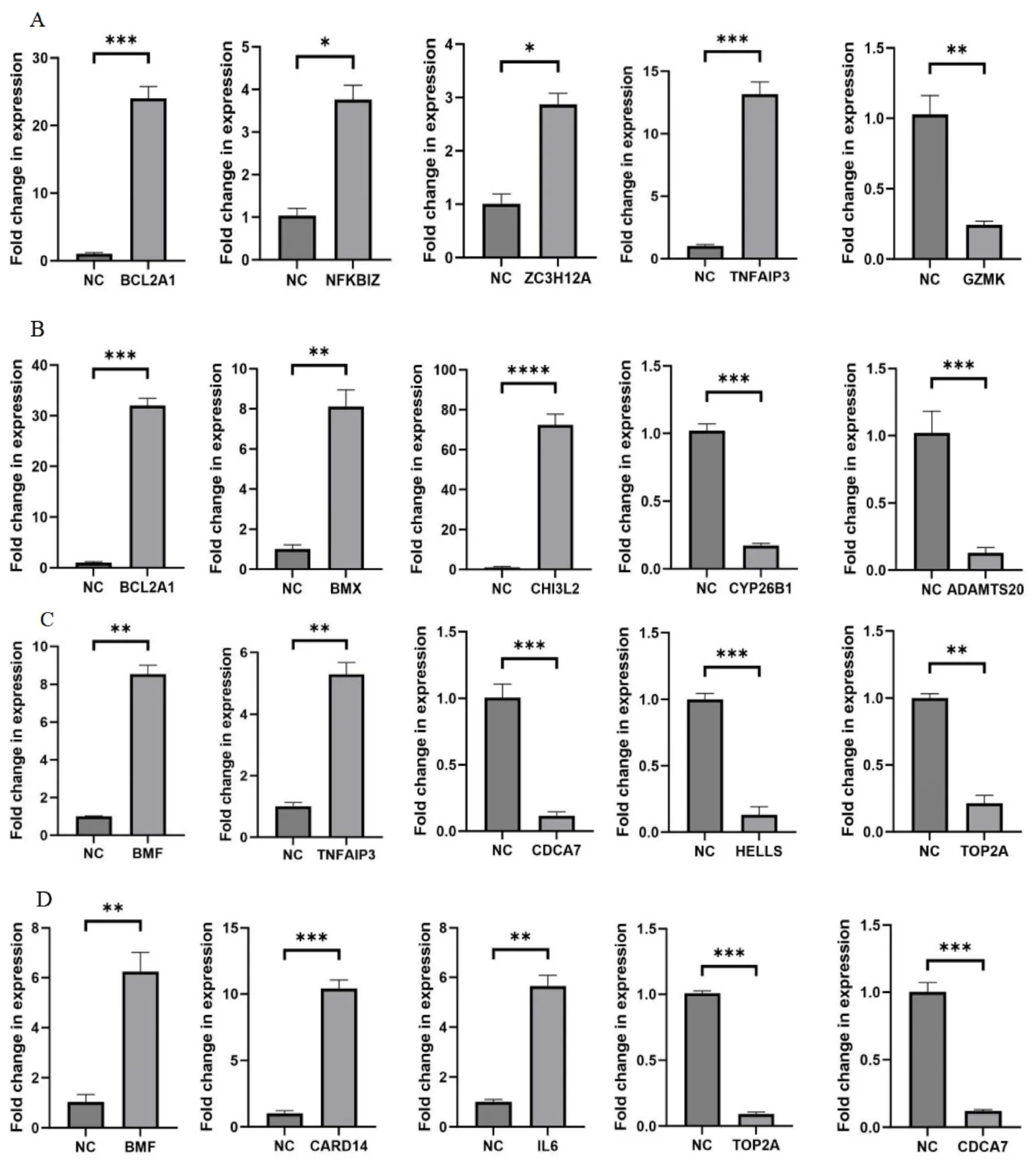
qRT-PCR validation of differential gene expression. Values are reported as mean ± SD based on three biological replicates (*n* = 3), with statistical significance evaluated by Student’s *t*-test (*p* < 0.05). Statistical significance was defined as follows: * *p* < 0.05; ** *p* < 0.01; *** *p* < 0.001; **** *p* < 0.0001. (**A**) 8 h; (**B**) 24 h; (**C**) 48 h; (**D**) 72 h.

**Figure 12:**
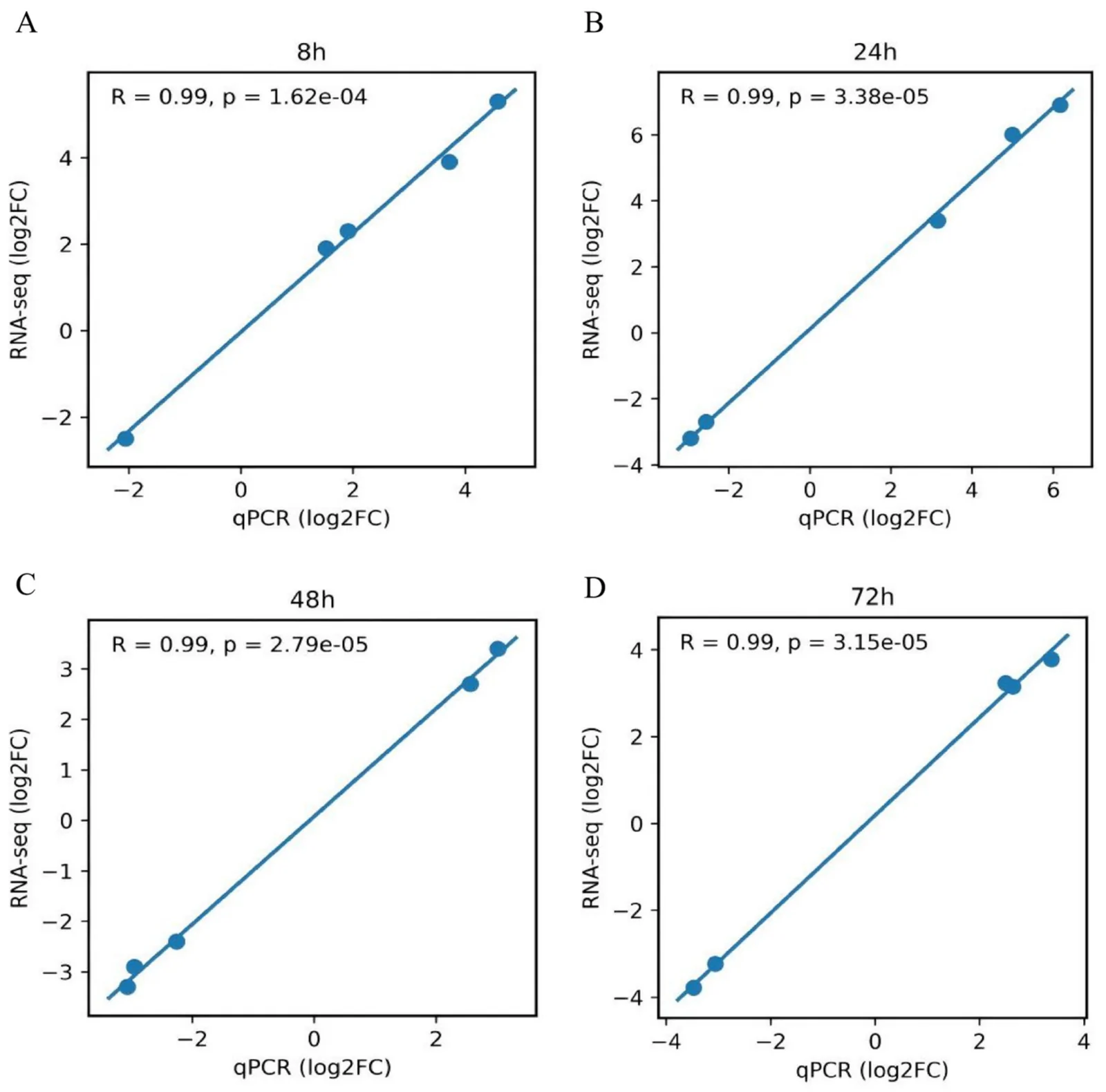
Association between RNA-seq and qRT-PCR results. The blue dots represent individual genes for correlation analysis. (**A**) 8 h; (**B**) 24 h; (**C**) 48 h; (**D**) 72 h.

The top five programmed cell death-related genes at each time point were selected for qRT-PCR validation using the corresponding RNA-seq samples, thereby assessing the reliability of the transcriptomic data (Figure 11). Pearson correlation analysis revealed significant positive correlations between the log_2_-transformed fold-change values obtained by qRT-PCR and RNA-seq at all time points (Figure 12). The correlation coefficient (*R*) was 0.99 at 8 h (*p* = 1.62 × 10^−4^), 0.99 at 24 h (*p* = 3.38 × 10^−5^), 0.99 at 48 h (*p* = 2.79 × 10^−5^), and 0.99 at 72 h (*p* = 3.15 × 10^−5^). The strong concordance between qRT-PCR and RNA-seq findings supported the reliability of the transcriptomic analysis.

## 4. Discussion

Our time-series transcriptomic and untargeted metabolomic analyses revealed sustained and dynamic remodeling of the host metabolic network following *M. bovis* infection of MAC-T cells, indicating that metabolic reprogramming is an early and persistent event in the host response to infection. At the early stage of infection (8 h), inflammatory signaling pathways, including NF-κB, TNF, and IL-17, were rapidly activated, whereas metabolic alterations were relatively limited. This pattern suggests that the host initially mounts an innate immune response centered on mammary epithelial cells and subsequently transitions toward metabolic adaptation. Mammary epithelial cells not only constitute a physical barrier within mammary tissue but also express multiple pattern-recognition receptors and secrete inflammatory mediators, thereby serving as important regulators of local mammary immune responses [37]. As infection progressed, the number of DAMs continued to increase, involving multiple metabolic networks, including amino acid, lipid, and nucleotide metabolism. These findings indicate a gradual transition from inflammatory activation toward extensive metabolic remodeling in host cells. This time-dependent shift from immune activation toward metabolic adaptation highlights the dynamic nature of the host response to *M. bovis* infection.

In recent years, immunometabolism has been recognized as an important regulatory mechanism underlying host responses to pathogen infection, with bidirectional interactions between inflammatory signaling and cellular metabolic networks representing a central feature of this process [38,39]. During bovine mammary infection, pathogens initially activate mammary epithelial cells and the local innate immune system upon entering the mammary gland. The intensity and duration of inflammatory responses vary considerably among pathogens, and these immune responses, in turn, further influence the metabolic state of host cells [13,40]. Pathogen stimulation can induce a metabolic shift in host cells resembling the Warburg effect. This shift involves the remodeling of glycolysis and related metabolic networks, thereby providing the energy and biosynthetic substrates required to support inflammatory responses [17]. Although glycolysis-related metabolites were not directly measured in the present study, the activation of inflammatory pathways and the sustained remodeling of metabolic networks exhibited highly synchronized temporal patterns. These findings suggest that *M. bovis* infection can likewise drive extensive immunometabolic reprogramming in host cells. Moreover, these metabolic alterations may not simply represent passive consequences of inflammatory activation but may instead constitute an important basis for sustaining the host inflammatory response and persistent pathogen infection. Changes in amino acid metabolism further support this view. In the present study, (S)-Leucic acid remained elevated at multiple stages of infection and showed consistent correlations with inflammation-related genes, including SAA3 and SGSH. Branched-chain amino acid metabolism not only provides energy substrates for cells but can also regulate immune cell activation and inflammatory cytokine expression through signaling pathways such as mTOR and NF-κB [41]. Thus, the gene–metabolite associations identified in this study suggest that remodeling of amino acid metabolism may contribute to host inflammatory adaptation and energy compensation, providing evidence of the coordinated interaction between immune signaling and metabolic regulation during infection. In addition to amino acid metabolism, nucleotide-related pathways, including pyrimidine and choline metabolism, were also persistently enriched. Nucleotide metabolism is involved not only in DNA repair, RNA synthesis, and cell proliferation but also in the regulation of host inflammatory responses through its effects on nucleic acid synthesis and energy metabolism [20,42]. Because *M. bovis* has limited metabolic capacity and relies heavily on the host for nucleotides and other metabolic substrates to support long-term survival [1,43,44], remodeling of the host metabolic network may represent both an adaptive response to pathogen invasion and a metabolic environment that can be exploited by the pathogen to maintain persistent infection. Consistent with the combined transcriptomic and metabolomic analyses, core genes such as SAA3 and SGSH showed significant associations with multiple DAMs, further indicating that inflammatory signaling, metabolic regulation, and host adaptation jointly constitute a dynamic regulatory network in the early stages of infection and establish the metabolic basis for subsequent redox imbalance and programmed cell death.

The continued progression of metabolic reprogramming further disrupts redox homeostasis in host cells, positioning oxidative stress as an important link between metabolic abnormalities and cellular injury. Earlier research has demonstrated that infection with *M. bovis* could induce sustained ROS accumulation in bovine mammary epithelial cells and promote apoptosis through activation of the mitochondria-dependent apoptotic pathway [16]. By integrating time-series multi-omics analyses, the present study further revealed that oxidative stress is not a transient response following infection but rather a dynamic process that progressively intensifies alongside the sustained remodeling of metabolic networks. This process is consistent with the characteristics of mammary infection, in which persistent activation of innate immunity leads to the continuous release of inflammatory mediators, thereby further driving tissue injury and amplifying oxidative stress [39]. As infection progressed, amino acid, lipid, and nucleotide metabolism underwent continuous remodeling, suggesting that sustained ROS accumulation is more likely a secondary consequence of progressive metabolic reprogramming than a direct product of inflammatory activation itself. Previous studies have shown that metabolic abnormalities can impair mitochondrial electron transport chain function, increase electron leakage, and promote continuous ROS production, thereby establishing a positive feedback loop that exacerbates oxidative stress [21,45]. Notably, the metabolomic analysis in the present study revealed pronounced time-dependent changes in glutathione (GSH): GSH levels decreased significantly at 8 h post-infection, transiently recovered at 24 h, subsequently declined to their lowest level at 48 h, and remained low at 72 h. This dynamic pattern indicates that host cells can initiate a degree of compensatory response by modulating antioxidant metabolism during the early stage of infection; however, as infection persists, intracellular GSH reserves are progressively depleted, ultimately leading to decompensation of the antioxidant system. This dynamic transition from “early compensation” to “late decompensation” represents an important finding of the present time-series analysis and further indicates that redox imbalance is a consequence of progressive infection rather than a stress response confined to a single time point. Because GSH is the essential reducing substrate required by GPX4 to eliminate lipid peroxides, its sustained depletion may impair the cellular capacity to remove lipid peroxides, thereby further amplifying oxidative damage [27,46]. In addition, the metabolomic analysis showed sustained enrichment of pathways related to unsaturated fatty acid biosynthesis, sphingolipid metabolism, and primary bile acid biosynthesis. The marked decrease in sphinganine suggested disruption of sphingolipid homeostasis and may have contributed to an imbalance in Ceramide/S1P signaling, thereby promoting inflammatory amplification, mitochondrial dysfunction, and ROS accumulation [47] and creating favorable conditions for ROS-mediated lipid peroxidation. Previous studies have confirmed that polyunsaturated fatty acids are major substrates targeted by ROS and that their oxidation products can further disrupt cell membrane integrity and amplify oxidative damage [48]. Integrated analysis further revealed significant associations between key genes, including SAA3, SGSH, TANC1, SREK1, and NAA15, and multiple DAMs. These findings indicate that inflammatory responses, metabolic reprogramming, and redox imbalance are not independent processes but instead jointly constitute a dynamically evolving gene–metabolite regulatory network during infection, providing a molecular basis for the subsequent development of programmed cell death processes, including apoptosis, pyroptosis, and ferroptosis.

As oxidative stress continues to intensify, host cells gradually transition from metabolic adaptation to irreversible injury, ultimately resulting in the activation of multiple programmed cell death pathways. Transcriptomic analysis in the present study showed sustained enrichment of apoptosis, cellular senescence, and the TNF signaling pathways during the late stages of infection, accompanied by suppression of pathways such as cell cycle and DNA replication. These transcriptomic alterations, together with changes in BMF, BCL2A1, and TOP2A expression, further suggest that *M. bovis* infection may be associated with impaired DNA replication, cell-cycle arrest, and mitochondrial dysfunction, which may collectively promote mitochondria-dependent apoptosis. Earlier research has demonstrated that *M. bovis* could trigger caspase-dependent apoptosis by inducing ROS accumulation and mitochondrial damage [16]. The present study further suggests that apoptosis may arise as a consequence of the sustained progression of metabolic imbalance and oxidative stress during infection, rather than as an independent cellular event. In addition to apoptosis, the sustained upregulation of inflammation-related genes, including NF-κB, TNF, IL6, and CARD14, suggested that inflammasome-mediated pyroptosis may also contribute to host cell injury during the late stages of infection. Previous studies have shown that persistent activation of NF-κB signaling can promote NLRP3 inflammasome assembly, subsequently activating caspase-1 and promoting IL-1β maturation and release, as well as Gasdermin D cleavage, ultimately leading to plasma membrane pore formation and pyroptosis [49,50]. Although key molecules such as NLRP3 and Gasdermin D were not examined in the present study, the sustained activation of inflammatory signaling suggests that pyroptosis may act together with apoptosis to contribute to inflammatory amplification and cellular injury during the later stages of infection. Notably, integrated multi-omics analyses provide correlative evidence suggesting the potential involvement of ferroptosis during *M. bovis* infection. Unlike previous studies that have primarily focused on apoptosis, the metabolomic analysis in the present study revealed significant enrichment of the ferroptosis pathway at 48 h, accompanied by sustained GSH depletion, enhanced unsaturated fatty acid biosynthesis, and a marked decrease in sphinganine. Collectively, these multi-omics observations point to a potential correlative association, yet they cannot confirm the actual occurrence of ferroptosis upon *M. bovis* infection. Ferroptosis is a form of programmed cell death driven by iron-dependent lipid peroxidation and is associated with GSH depletion, impaired GPX4 antioxidant activity, and sustained oxidation of polyunsaturated fatty acids [51,52]. In the present study, GSH exhibited a dynamic pattern characterized by transient recovery at the early stage followed by sustained depletion at later stages, while earlier research has demonstrated that *M. bovis* infection could induce persistent ROS accumulation [16]. Together with the enhanced unsaturated fatty acid metabolism and disrupted sphingolipid metabolism observed in the present study, these findings suggest that host cells develop a redox environment conducive to the sustained accumulation of lipid peroxidation during the later stages of infection. This metabolic profile suggests a cellular state that may be conducive to ferroptosis. Previous studies have shown that ACSL4 plays a key role in the esterification of polyunsaturated fatty acids and in the remodeling of membrane phospholipid composition, and is also a central regulator of cellular sensitivity to ferroptosis [53]. Because GPX4, SLC7A11, ACSL4, and indicators of iron homeostasis were not examined in the present study, direct experimental evidence confirming the occurrence of ferroptosis remains lacking. Nevertheless, the enrichment of the ferroptosis pathway, GSH depletion, lipid metabolic remodeling, and enhanced oxidative stress together provide converging correlative multi-omics evidence that imply the potential involvement of ferroptosis in *M. bovis* infection. To the best of our knowledge, this study is the first to propose, based on integrated time-series transcriptomic and metabolomic analyses, that ferroptosis may contribute to mammary epithelial cell injury during *M. bovis* infection, thereby providing a new theoretical basis and research direction for subsequent investigation of its molecular mechanisms and functional validation.

In summary, using time-series transcriptomic and untargeted metabolomic analyses, we systematically characterized the dynamic molecular response of MAC-T cells to *M. bovis* infection and revealed a progressive pathological process extending from metabolic reprogramming and redox imbalance to the activation of multiple programmed cell death pathways. Based on these observations, we propose a potential pathogenic model linking metabolic reprogramming, oxidative stress, lipid peroxidation, and programmed cell death. Unlike previous studies that have primarily focused on inflammatory responses or individual forms of cell death, our study, to the best of our knowledge, represents the first time-series multi-omics study suggesting the potential involvement of ferroptosis in the pathogenic process of *M. bovis* infection. These findings expand our understanding of host responses to *M. bovis* infection and provide a theoretical basis for further investigating potential interventions targeting metabolic dysregulation and oxidative stress during *M. bovis*-associated mastitis. In the medium term, the metabolic and redox alterations identified in this study may help researchers prioritize candidate molecular targets and intervention strategies for further evaluation. If subsequently supported by functional and in vivo studies, these candidate targets may contribute to the development of preventive or therapeutic approaches aimed at reducing cellular injury and improving the management of *M. bovis*-associated mastitis in dairy cattle. Nevertheless, the mechanistic associations identified in our study were primarily inferred from time-series multi-omics analyses and require further validation through functional experiments. Future studies should further assess ROS accumulation, lipid peroxidation, iron levels, and key ferroptosis-related markers, including GPX4, SLC7A11, and ACSL4, and combine these analyses with ferroptosis inhibitor treatment and genetic intervention experiments [54] to determine whether and how ferroptosis contributes to *M. bovis* infection-induced cellular injury.

## 5. Conclusions

This study used time-series transcriptomic and untargeted metabolomic analyses to systematically characterize the dynamic host response of MAC-T cells to *M. bovis* infection, revealing sustained, time-dependent metabolic reprogramming throughout the course of infection. During infection, the activation of inflammatory signaling was closely coupled with the remodeling of amino acid, lipid, and nucleotide metabolism. This process was accompanied by progressive GSH depletion, disruption of sphingolipid metabolism, and increasing oxidative stress, suggesting that metabolic reprogramming and redox imbalance may reinforce one another and jointly contribute to host cell injury. Integrated multi-omics analysis further showed that the progressive intensification of oxidative stress was closely associated with the activation of pathways related to apoptosis, pyroptosis, and ferroptosis. In particular, the enrichment of the ferroptosis pathway together with sustained GSH depletion and lipid metabolic remodeling constituted a multi-omics evidence chain suggesting the potential involvement of ferroptosis in *M. bovis* infection. To the best of our knowledge, this study is the first to propose, based on integrated time-series transcriptomic and untargeted metabolomic analyses, that ferroptosis may contribute to mammary epithelial cell injury induced by *M. bovis* infection and to propose a dynamic pathogenic model characterized by “inflammatory activation–metabolic reprogramming–oxidative stress–lipid peroxidation–programmed cell death.” This model provides a theoretical framework for further understanding the mechanisms underlying host–*M. bovis* interactions. Further functional validation of ROS accumulation, lipid peroxidation, iron homeostasis, and key molecules such as GPX4, SLC7A11, and ACSL4 is required to clarify the regulatory mechanisms underlying ferroptosis during *M. bovis* infection and to provide a theoretical basis for developing prevention and control strategies targeting metabolic reprogramming and oxidative stress.

## Data Availability

The transcriptomic data obtained in this study is available in the NCBI Sequence Read Archive (SRA) database under accession number PRJNA1505148, while the metabolomics dataset has been submitted to the MetaboLights database under accession number MTBLS15252. Both datasets can be accessed publicly through their respective databases.

## Supplementary Materials

The following supporting information can be downloaded at: https://www.mdpi.com/article/10.3390/microorganisms14102241/s1. Table S1: qRT-PCR primer sequences.

## Abbreviations

The following abbreviations are used in this manuscript:

*M**. bovis*	*Mycoplasmopsis bovis*
MAC-T	Mammary alveolar cell-T
MOI	Multiplicity of infection
BP	Biological process
CC	Cellular component
MF	Molecular function
cDNA	Complementary DNA
DAMs	Differentially abundant metabolites
DEGs	Differentially expressed genes
GO	Gene Ontology
KEGG	Kyoto Encyclopedia of Genes and Genomes
KO	KEGG Orthology
LC-MS	Liquid chromatography–mass spectrometry
RNA-seq	RNA sequencing
NCBI	National Center for Biotechnology Information
OPLS-DA	Orthogonal partial least squares-discriminant analysis
PCA	Principal component analysis
PCD	Programmed cell death
qRT-PCR	Quantitative real-time polymerase chain reaction
HMDB	Human Metabolome Database
log_2_FC	Log_2_ fold change
VIP	Variable importance in projection
SD	Standard deviation
UHPLC	Ultra-high-performance liquid chromatography
ROS	Reactive oxygen species
GSH	Glutathione
SRA	Sequence Read Archive
