# Supplementary material for: Time-Series Multi-Omics Reveal Metabolic Reprogramming, Oxidative Stress and Programmed Cell Death in M. bovis-Infected Bovine Mammary Epithelial Cells
Source: Microorganisms. 2026 Oct 3;14(10):2241. doi: 10.3390/microorganisms14102241 (PMC13707408; doi:10.3390/microorganisms14102241)
Supplement: Supplementary file 1 [file microorganisms-14-02241-s001.zip › microorganisms-4525475-supplementary.pdf]

Table S1. qRT-PCR primer sequences.

Gene	Primer Sequence (5‘ → 3’)	Size (bp)
BCL2A1	F: ACTGGGGCAGGATTGTAACC<br>R: CAAACCCATTTTCCCAGCCTC	180
TNFAIP3	F: CACACACCCCCAGAATCCTC<br>R: ACACAAAGGCTGGGTGTTCA	115
GZMK	F: ACTGGGGCAGGATTGTAACC<br>R: ATGGGGCCAAGTTGCTTTTG	162
ZC3H12A	F: ACTGGGGCAGGATTGTAACC<br>R:GTCCTTGTTCCCATGGCTCA	204
NFKBIZ	F: ATTTTACTGGAAACCGGGCG<br>R:CCGGAGTGCCCGACTTTTTA	155
CHI3L2	F: ACTGGGGCAGGATTGTAACC<br>R:CAGTTTGGGATTCCCTGGGTG	217
BMX	F:AAAAACACGGGGTTCCCATCT<br>R:ATCTTCACTGCTTCTCAGTTTTCT	221
CYP26B1	F:CTTTGAAGGCTTGGAGCTGGT<br>R:CTTTGAAGGCTTGGAGCTGGT	215
ADAMTS20	F:CACTGGCCTCCTACGAAGTG<br>R:AGTGGGTTCTGAACGGTGTG	132
BMF	F:TTATGGCCATGCTGGCTACC<br>R:TCGGTTCTGCTGGTATTGCTG	181
CDCA7	F:CGCGGCAAAAGGATTGCAG<br>R:GTGCTTCGAGGGGGAAACTT	226
HELLS	F:GAAGATGCTAGAAAAGGCTCGC<br>R:CCTCTAACTGCTGCTGTTCCAT	140
TOP2A	F:GGAGAGCAGCAGCAAAAACC<br>R:AGGCACTCTTCAGTGGCTTC	171
CARD14	F:CTCATCAGAGCACCGTGACC<br>R:AAGCAGCTCTCGGTCCAAAA	187
IL6	F:CCAGCCACAAACACTGACCT<br>R:CCCCAGCTACTTCATCCGAA	141
β-actin	F:TCCATCGTCCACCGCAAATGCT<br>R:TGCTGTCACCTTCACCGTTCCA	198
